# COVID-19-Related Lung Involvement at Different Time Intervals: Evaluation of Computed Tomography Images With Semiquantitative Scoring System and COVID-19 Reporting and Data System Scoring

**DOI:** 10.7759/cureus.18554

**Published:** 2021-10-06

**Authors:** İnan Korkmaz, Fatma Keleş

**Affiliations:** 1 Department of Radiology, Hatay Mustafa Kemal University, Faculty of Medicine, Antakya, TUR

**Keywords:** atypical findings, co-rads, ct severity score, chest ct, covid-19

## Abstract

Introduction: This study aimed to evaluate the frequency of typical and atypical thoracic CT findings in patient groups diagnosed during different periods of the pandemic, examine disease severity using radiological scoring methods, and determine the relationship between atypical CT findings and disease severity.

Materials and methods: One hundred fifty-one patients with positive reverse transcription polymerase chain reaction (RT-PCR) test and thoracic CT scan were included in the study. The patients were divided into two groups as group 1 (March to August 2020) diagnosed in the first six months of the pandemic and group 2 (September 2020 to February 2021) diagnosed in the second six months. CT images of the patients were analyzed for the frequency of typical and atypical findings. Evaluation was made in terms of disease suspicion and severity by scoring methods, and the relationship between atypical findings and disease severity was examined.

Results: There was no statistically significant difference between the frequency and distribution patterns of typical CT findings observed in both groups. The most common atypical finding in both groups was nodular lesions. Central distribution, one of the atypical findings, was not seen in group 1, whereas it was present in nine patients in group 2 (p=0.001). The mean CT severity score was higher in group 2, and there was a statistically significant difference between the mean CT scores of both groups (p<0.001). In addition, six (7.2%) patients in group 1 and 34 (50%) patients in group 2 had CT scores above the cut-off value (p<0.001). There was no statistically significant relationship between atypical findings and severity score.

Conclusion: Other diseases and atypical findings that may accompany COVID-19 pneumonia may increase the rate of misdiagnosis. In the diagnosis of the disease, clinical signs and symptoms and radiological findings should be evaluated together, and it should be kept in mind that lung findings in thorax CT change over time.

## Introduction

In December 2019, a new coronavirus named severe acute respiratory syndrome coronavirus 2 (SARS-CoV-2), which was pointed as the cause of coronavirus disease 2019 (COVID-19), was identified [1]. Since the World Health Organization declared it a pandemic, the COVID-19 outbreak continues to put pressure on societies worldwide.

It was understood that the clinical findings of the disease vary from symptoms such as fever, myalgia, and headache, which can also be seen in other viral infections, to pneumonia that can cause severe respiratory failure and death [2,3].

It was understood that the reverse transcription polymerase chain reaction (RT-PCR) test, which is the gold standard in diagnosis, showed false negatives due to different reasons. Studies reported that the sensitivity of the test varies between 42% and 83% [4-7]. Moreover, it is known that the RT-PCR test, which is negative at the beginning, can turn positive in retests [8,9]. Therefore, thorax computed tomography (CT) examination has gained importance and has started to play a role in diagnosing, triage, and determining disease severity [5,8,10].

Typical CT findings of COVID-19 pneumonia are ground-glass opacities and consolidations, mainly displaying lower lobe and peripheral distribution. In addition to these findings, crazy paving pattern, vascular enlargement, air bubble sign, halo and reverse halo signs, airway changes, and air bronchogram are less common findings [11-14]. Central distribution, peribronchovascular spread, isolated upper lobe involvement, lobar and segmental consolidation, nodules, subpleural sparing, pleural and pericardial effusion, tree-in-bud pattern, and white lung can be seen as atypical findings [1,15,16].

Since atypical findings can also be seen in other viral infections and lung diseases and reporting these findings may cause false-negative cases, radiology associations and radiologists have developed scoring systems like the COVID-19 Reporting and Data System (CO-RADS) and CT severity score (CT-SS) to make an objective assessment and predict the severity of the disease [4,17-19].

This study aimed to evaluate the frequency of typical and atypical thoracic CT findings in patient groups diagnosed during different periods of the pandemic, examine disease severity using radiological scoring methods, and determine the relationship between atypical CT findings and disease severity.

## Materials and methods

The local ethics committee approved this retrospective study, and the requirement for informed consent was waived (11/03/2021, meeting number 4, decision number 26). The study protocol complies with the ethical guidelines of the 1975 Declaration of Helsinki, which the institution's human research committee previously approved.

Patients

Throughout the year from March 2020, when the first COVID-19 case was seen in our country, to February 2021, 280 patients who applied to our hospital with the suspicion of COVID-19 infection complained of shortness of breath and had RT-PCR test were retrospectively analyzed.

Exclusion Criteria

Patients with negative initial RT-PCR test or not positive in repeat tests, patients with positive RT-PCR test but no CT examination, patients with known lung disease and history of lobectomy, patients younger than 18 years of age, and pregnant women were excluded. One hundred twenty-nine patients with these criteria were excluded from the study.

As a result, a total of 151 patients who had a positive RT-PCR test and underwent thoracic CT scan due to shortness of breath or low oxygen saturation at the first admission were included in the study.

Due to severity and intensity of thorax CT imaging findings of COVID-19 patients increased over time during the pandemic process and due to the fact that atypical CT findings have begun to be defined in the literature, we aimed to evaluate the frequency of typical and atypical CT findings and the relationship of these findings with the severity of the disease at different time intervals. As a result, the patients were divided into two groups. Group 1 included patients diagnosed within the first six months of the pandemic (March to August 2020), and group 2 included patients diagnosed within the second six months (September 2020 to February 2021) of the pandemic. Thoracic CT findings of the patients in the groups were examined.

Since our study was to examine the typical and atypical findings on thorax CT images obtained at the time of first admission in COVID-19 patients and to investigate the relationship between CT severity score and atypical findings, the patients were not classified according to their clinical conditions and their treatment processes were not evaluated. Since the RT-PCR test is the gold standard in diagnosis, patients who did not show a positivity in repeat tests were not included in the study.

CT protocol

CT scans were performed in the supine position using either the Hitachi Eclos 16 (Hitachi Medical Corporation, Japan; 5-mm slice thickness, 120 kV, 75 mAs) or 64-slice Toshiba Aquilion (Toshiba Medical System Corporation, Model TSX-101A, Otawara-Shi, Japan; 5-mm slice thickness, 120 kV, 25 mAs) units. Images were acquired in the supine position during deep inspiration. Multiplane images were obtained using the multiplane reformatting (MPR) technique on a workstation.

Image analysis

Two radiologists (with 9 and 10 years of experience) reviewed the CT images and made a consensus decision. Due to the current known outbreak and the retrospective nature of our study, evaluators could not be blinded to patients being COVID-19 positive.

Typical CT findings were categorized as peripheral ground-glass opacities and/or consolidation, crazy paving pattern, airway changes, halo sign, reverse halo sign, and air bubble sign.

Central distribution, peribronchovascular spread, isolated upper lobe involvement, lobar and segmental consolidation, nodular lesions, subpleural sparing, pleural and pericardial effusion, tree-in-bud pattern, and white lung were evaluated as atypical CT findings.

CO-RADS according to CT findings

The level of suspicion for COVID-19 pneumonia was evaluated with the CO-RADS scoring [4]. CO-RADS 1: COVID-19 is highly unlikely, CT is normal, or noninfectious findings; CO-RADS 2: the level of suspicion of COVID-19 infection is low, and findings consistent with a different infection; CO-RADS 3: COVID-19 infection is unsure or indeterminate, and features in CT that may be compatible with COVID-19 and other diseases; CO-RADS 4: the level of suspicion is high, and suspicious CT findings for COVID-19 but not extremely typical; and CO-RADS 5: the level of suspicion is very high, and typical CT findings.

CT severity score

We calculated the CT-SS to evaluate the involvement rate of the lungs. CT-SS was calculated according to the degree of lobar involvement. Eighteen segments of both lungs were divided into 20 regions. The apicoposterior segment of the left upper lobe was apical and posterior. The anteromedial segment of the left lower lobe was anterior and basal. Each region was visually scored on a 0 to 2 numerical scale. No involvement (0%) was scored as '0', involvement less than 50% was scored as '1', and involvement equal to or more than 50% was scored as '2' [17]. The total CT score, which could range from 0 to 40, was calculated for each patient. CT scores of the groups were calculated as mean ± SD and statistically evaluated. The relationship between the atypical CT findings in the groups and the severity score was examined.

Statistical analysis

In our study, the data were analyzed using the SPSS 23 program (IBM SPSS Statistics for Windows, Version 23.0, Armonk, NY: IBM). Quantitative data were defined as mean ± standard deviation. The frequency of radiological findings and the number of occurrences in each cluster were expressed as a percentage. A comparison of radiological findings and distributions between groups was made with the Mann-Whitney U test. Independent samples T-test was used for correlation of CT-SS and age between groups. Pearson Chi-Square test was used to evaluate the relationship between atypical findings and CT-SS. The limit of significance was accepted as 0.05 for all tests. 

## Results

Of the 151 patients included in the study, 63 (41.7%) were female, and 88 (58.3%) were male. The mean age of all patients was 52.77 ± 17.3, and the age range was between 18 and 92. When the distribution according to the groups was examined, there were 35 (42.2%) women and 48 (57.8%) men in group 1 and 28 (41.2%) women and 40 (58.8%) men in group 2. The mean age of group 1 was 47.88 ± 16.8 (range: 18-92), and the mean age of group 2 was 58.75 ± 16.1 (range: 25-92). There was a statistically significant difference between the two groups according to the mean age (p<0.001).

Imaging findings

Consistent with the literature, the most common lesion patterns in thorax CT examinations of patients were ground-glass opacity in 62 patients (41.1%) and ground-glass opacity and consolidation in 49 patients (32.5%); 24 (15.9%) patients (20 in group 1 and four in group 2) had no involvement. In addition to this finding, crazy paving pattern in 10 patients (6.6%), airway change in four patients (2.6%), reverse halo sign in six patients (4%), halo sign in three patients (2%), and air bubble sign in three patients (2%) were accompanied.

**Figure 1:**
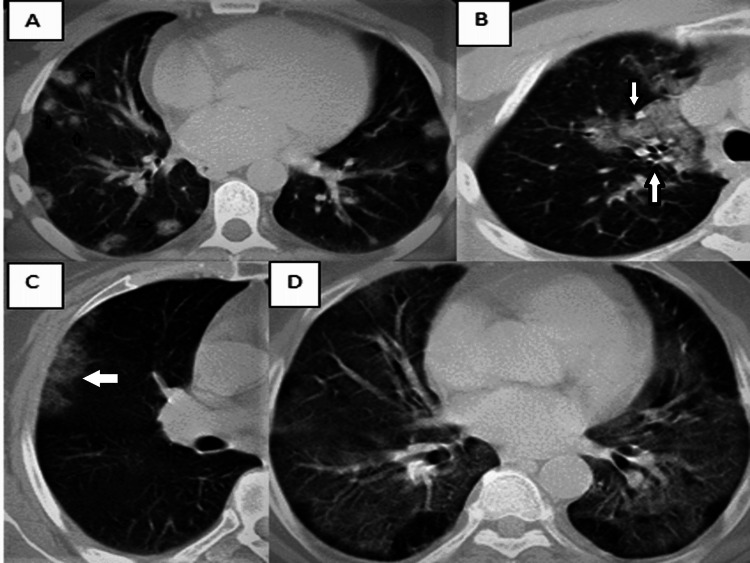
Examples of atypical findings observed in patients A. Nodular involvement is observed in a 39-year-old male patient in group 1 (small arrows). B. Axial CT image of a 55-year-old man in group 2 shows peribronchovascular involvement in the right upper lobe (white arrows). C. Peripheral ground-glass opacity is observed in the CT image of a 60-year-old female patient in group 1 with isolated upper lobe involvement (white arrow). D. A 70-year-old male patient in group 2 with white lung.

When atypical CT findings are examined, nodular involvement in 28 patients (18.5%), central distribution in nine patients (6%), pleural effusion in five patients (3.3%), segmental consolidation in five patients (3.3%), subpleural sparing in five patients (3.3%), and white lung in five patients (3.3%), lobar consolidation in four patients (2.6%), peribronchovascular spread in four patients (2.6%), tree-in-bud pattern in four patients (2.6%), pericardial effusion in two patients (1.3%), and isolated upper lobe involvement (1.3%) in two patients were present (Figure 1).

There was a peripheral distribution pattern in 73 patients (48.3%) and a peripheral and central distribution pattern in 45 patients (29.8%). Common lower lobe involvement was most frequently observed [right lower lobe: 109 (72.2%) and left lower lobe: 109 (72.2%)]. Tables 1, 2 show the distribution of typical and atypical findings, distribution patterns, and sites of involvement in thorax CT scans by groups.

**Table 1:** Typical and atypical findings on CT images GGO: ground-glass opacity.

	Group 1 (n=83)	Group 2 (n=68)	p
Typical findings			
GGO	23 (27.7%)	39 (42.6%)	<0.001
GGO and consolidation	36 (43.4%)	13 (19.1%)	0.002
Crazy paving pattern	3 (3.6%)	7 (10.3%)	0.102
Air bubble sign	3 (3.6%)	0 (0.0%)	0.114
Halo sign	1 (1.2%)	2 (2.9%)	0.448
Reversed halo sign	6 (7.2%)	0 (0.0%)	0.024
Airway changes	2 (2.4%)	2 (2.9%)	0.840
Atypical findings			
Nodule	19 (22.9%)	9 (13.2%)	0.130
Central distribution	0 (0.0%)	9 (13.2%)	0.001
Pleural effusion	2 (2.4%)	3 (4.4%)	0.495
Segmental consolidation	1 (1.2%)	4 (5.9%)	0.111
Subpleural sparing	5 (6%)	0 (0.0%)	0.040
White lung	1 (1.2%)	4 (5.9%)	0.111
Lobar consolidation	1 (1.2%)	3 (4.4%)	0.224
Peribronchovascular involvement	2 (2.4%)	2 (2.9%)	0.840
Tree-in-bud pattern	2 (2.4%)	2 (2.9%)	0.840
Pericardial effusion	1 (1.2%)	1 (1.5%)	0.887
Isolated upper lobe involvement	2 (2.4%)	0 (0.0%)	0.199

CO-RADS assessment

Although the RT-PCR positivity of all patients was known due to the retrospective nature of our study, in the examination made on imaging findings, there were a total of 47 (31%) patients, 25 in group 1 and 22 in group 2, in CO-RADS categories 1, 2, and 3 that could be considered false negative for COVID-19 pneumonia. One hundred four patients were in CO-RADS categories 4-5, suspicious for COVID-19 pneumonia or consistent with typical findings.

**Table 2:** Lesion distribution patterns on CT images

	Total (n=151)	Group 1 (n=83)	Group 2 (n=68)	p
Distribution pattern				
Peripheral	73 (48.3%)	35 (42.2%)	38 (55.9%)	0.094
Peripheral and central	45 (29.8%)	28 (33.7%)	17 (25%)	0.245
Distribution				
Bilateral	109 (72.2%)	53 (63.9%)	56 (82.4%)	0.012
Right upper lobe	94 (62.3%)	42 (50.6%)	52 (76.5%)	0.01
Right middle lobe	81 (53.6%)	35 (42.2%)	46 (67.6%)	0.02
Right lower lobe	109 (72.2%)	53 (63.9%)	56 (82.4%)	0.012
Left upper lobe	98 (64.9%)	46 (55.4%)	52 (76.5%)	0.007
Left lower lobe	109 (72.2%)	53 (63.9%)	56 (82.4%)	0.012

CT severity score assessment

**Figure 2:**
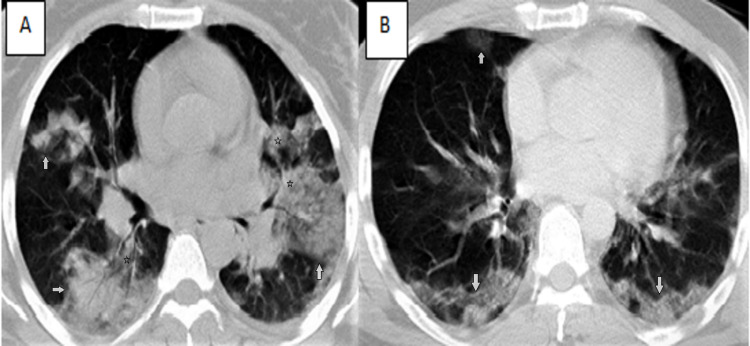
CT images of patients with high CT-SS A. The CT image of a 42-year-old female patient (in group 2) with bilateral involvement in all lobes shows central (stars) and peripherally (white arrows) distributed ground-glass opacities and consolidated areas containing air bronchograms (CT-SS:24). B. Axial CT image of a 45-year-old male patient (in group 2) with CT-SS:22 shows bilateral peripheral ground-glass opacities (white arrows). CT-SS: CT severity score.

In our study, the total CT score was obtained by summing the involvement scores of all segments in both lungs, and the score ranged from 0 to 40 in each patient. The mean CT score was 7.79 ± 7.87 in group 1 and 18.52 ± 11.4 in group 2, and there was a very high statistically significant difference between the mean CT score of both groups (p<0.001). In their examination of the severity of the disease, Yang et al. determined the cut-off value of the CT score for severe disease as 19.5 [17]. In our study, the CT score was above 19.5 in six (7.2%) patients in group 1 and above 34 (50%) patients in group 2; in the examination, we performed by taking the cut-off value of 19.5 (p<0.001) (Figure 2).

In our study, no statistically significant correlation was found between atypical CT findings and severity score when examined individually in our evaluation of CT-SS in patients with atypical findings in both groups.

## Discussion

COVID-19 pneumonia is similar to severe acute respiratory syndrome (SARS) and Middle East Respiratory Syndrome (MERS) [20,21] pneumonia, with ground-glass opacities and consolidative lesions predominant in peripheral areas and often bilaterally distributed, but the effect has been much longer and global. Different treatment options have been tried since the beginning of the pandemic [22-24], but the definitive treatment has not yet been clarified. The severity of the disease affects the treatment options, and thoracic CT, which detects the lung findings of the disease and evaluates the severity of the illness with scoring studies, has gained importance [10].

With many studies, the typical findings of COVID-19 pneumonia have been clarified, and the most common results are peripherally distributed ground-glass densities and/or consolidations [25,26]. In this study, in which we compared the severity of the disease using typical and atypical thoracic CT findings and radiological scoring methods in the patient groups diagnosed at different periods of the pandemic, the most common lesion patterns in both groups were ground-glass opacities and/or consolidations, which is consistent with the literature.

Studies conducted during the pandemic period have begun to describe various atypical findings in addition to typical and diagnostic CT findings in COVID-19 patients [16]. These findings have gained importance due to the increase in the incidence of the atypical findings in the COVID-19 patients, these findings can also be seen in other pulmonary infections and diseases accompanying COVID-19, and the atypical features may cause false-negative cases [10]. In our study, central distribution, one of the atypical findings, was not seen in group 1, whereas it was present in nine patients in group 2 (p=0.001). This finding may cause false-negative diagnoses in the bronchovascular distribution pattern of organizing pneumonia and in patients with concomitant heart failure [15]. The most common atypical finding in both groups was nodular lesions (19 patients in group 1 and nine patients in group 2). Although the incidence of nodules in COVID-19 pneumonia was reported to be between 3% and 13% [27] in the literature, it was found to be 18.5% in our study. There was no statistically significant difference in the diversity and incidence of other atypical findings in both groups.

Considering the distribution and distribution patterns, both groups had bilateral peripheral lower lobe dominance, and consistent with the literature, there was no statistically significant relationship between the groups.

Various studies were conducted to standardize CT reports in patients with suspected COVID-19, as CT findings partially similar to different viral infections and pulmonary diseases can be seen in COVID-19 pneumonia. One of these is the CO-RADS classification, which causes a level of suspicion for the pulmonary involvement of COVID-19 based on the features seen on chest CT [4]. This classification uses a 0 to 5 scoring system to classify signs of lung involvement from the normal/noninfectious category to the category highly/very highly suspected for COVID-19. In our study, 47 patients (31%) had a false-negative CT result for COVID-19 pneumonia (CO-RADS ≤ 3), based on imaging findings. Twenty-five of these patients were in group 1, and 22 were in group 2, and there was no statistically significant difference between the groups. There was no lung involvement in 25 patients from both groups (CO-RADS: 1). Studies reported that ground-glass opacities usually appeared on follow-up CT scans within the first five days after the onset of symptoms and reached their peak level (GGO and consolidation) approximately 6-14 days after the onset of symptoms [28,29]. It was found out that chest imaging may result negative in these patients in the early stages of the disease [9,17].

Our study performed a semiquantitative CT scoring to assess the extent and severity of COVID-19 pneumonia. As a result of the examination performed after both lungs were divided into 20 segments, the scores of the patients ranged from 0 to 40. The mean CT-SS was higher in group 2, and there was a statistically significant difference between the mean CT scores of both groups (p<0.001). Yang et al. found the cut-off value for CT score as 19.5 for severe disease [17], and we took the cut-off value as 19.5 for severe disease in our study. Accordingly, the CT score was above 19.5 in six (7.2%) patients in group 1 and in 34 (50%) patients in group 2 (p<0.001). Our study showed a statistically significant difference between the mean age of the patients in the groups, and the higher CT-SS in group 2 may be related to this. However, when the patients are examined individually, it is observed that the CT-SS is higher in the younger patients in group 2. Since our study is a study on CT findings of patients, clinical and laboratory findings were not evaluated. We think that this difference between CT-SS may be due to the fact that patients go to health institutions after the clinical symptoms worsen and their hospitalization is delayed in the later stages of the pandemic compared to the early stages of the pandemic [28,29].

In a study where Sahin et al. compared the patient groups with typical and atypical findings by taking the cut-off value as 7.5 with a different scoring method, it was reported that the CT-SS was significantly higher in the group with atypical results [30]. In our study, there was no statistically significant relationship between atypical findings and severity score in our examination of CT-SS in patients with atypical results in both groups. In addition, nodular lesions, the most common atypical finding in our study, were more common in patients with severity scores below the cut-off value in both groups (94.7% in group 1 and 77.8% in group 2).

Our study had some limitations. First of all, due to the retrospective nature of our research, the readers knew that the RT-PCR test of the patients resulted positive, and the CO-RADS evaluation was conducted according to the CT findings based on consensus. Secondly, since the CT findings of the patients at the time of the first admission were evaluated, the clinical and laboratory findings of the patients and any additional findings that may have emerged during the follow-up were not assessed. Apart from these, it was thought that the CT-SSs of the patients in the group 2 might be due to viral mutations. However, no examination was made for the mutations in our hospital at that time. Thirdly, patients were not classified according to their clinical status and posttreatment changes were not evaluated. Finally, a CT score assessment was performed by two relatively experienced radiologists. More research is needed to determine the degree of consistency of CT-SS among readers with different experience levels.

## Conclusions

Radiological imaging and especially thoracic CT have an important place in the diagnosis of COVID-19 pneumonia. Radiologists need to recognize the typical and atypical chest CT findings of COVID-19. Although we did not find a significant relationship between atypical findings and disease severity in our study, it should be kept in mind that other diseases and atypical findings that may accompany COVID-19 pneumonia may increase the rate of misdiagnosis. More imaging-based studies are needed to examine the relationship between atypical findings in COVID-19 pneumonia and disease severity.

In addition, our findings show that there is an increase in the CT severity scores of the patients, independent of typical and atypical findings, in the patient group diagnosed in the later stages of the pandemic. In COVID-19 patients, clinical findings and radiological findings should be evaluated together in terms of the progression of the disease, and it should not be forgotten that lung findings in thorax CT may change over time.
